# Clinical and Diagnostic Considerations for Atypical, Adult Onset Presentation of Chronic Recurrent Multifocal Osteomyelitis (CRMO)

**DOI:** 10.1155/2019/8206892

**Published:** 2019-09-29

**Authors:** Stacey Mahady, Amit Ladani

**Affiliations:** West Virginia University Medicine, 1 Medical Center Drive, Morgantown, WV 26506, USA

## Abstract

Chronic recurrent multifocal osteomyelitis (CRMO) is the most severe form of chronic nonbacterial osteomyelitis (CNO) and is a rare autoinflammatory bone disorder that mostly affects children and adolescents. CRMO is a diagnosis of exclusion, resulting in often-delayed diagnosis with over one year on average from onset of symptoms to time of diagnosis. Initial diagnosis is rare in adults and previously undocumented in the elderly (age greater than 65). We highlight a case of a 74-year-old elderly Caucasian female with a history of palmoplantar pustular psoriasis who presented with pelvic and hip pain. Imaging findings included multiple bony lesions on x-rays, increased uptake in the left side of the pelvis, ileum, proximal sternum, and bilateral medial clavicles on nuclear bone scan. Bone biopsy histologic results of marrow fibrosis and plasma cell infiltrate indicative of chronic inflammation lead to the diagnosis of CRMO. This case highlights that while CRMO is typically a disease with childhood onset, it, while rare, can also present in adults and now has presented in the elderly, remaining an important part of the differential diagnosis of bone pain in adults and the elderly in addition to infectious osteomyelitis and malignancy when imaging reveals multiple bony lesions. This in turn will facilitate the reduction of unnecessary medical treatment and antibiotics.

## 1. Introduction

Chronic recurrent multifocal osteomyelitis (CRMO) is a rare, inflammatory skeletal disease of unknown origin, which mainly affects children and adolescents in terms of cleido-spondylo-metaphysal skeletal inflammation. Only 10% of the patients are older than 20 years. Review of literature is scarce for documentation of CRMO in adults greater than 50 years of age. The largest reported cohort of CNO patients found is a 486-case series from the Eurofever international registry across 19 countries which documents 31 adult patients with a mean age of onset of 33 years (range 19–62.4 years) [1]. Thus, our patient likely appears to be the first documented case of CRMO onset in an elderly patient greater than 65 years of age. CRMO was first described by Giedion et al. in 1972 [2] and is characterized by sterile, multifocal, recurrent bone lesions related to aseptic osteoarthritis. Patients may typically present with multifocal painful osseous localizations [3]; however, clinical diagnosis is challenging as the presenting symptoms and course of disease is quite variable. The metaphyses of the long bones, pelvis, and clavicles are the most commonly affected sites, the vertebrae and mandible are also frequently involved [3], and the neurocranium is almost never affected [4]. Recent studies have described this autoinflammatory entity in concurrence with other autoimmune diseases such as vasculitis, ulcerative colitis, Crohn's disease, plantar pustulosis, psoriasis, acne, and Sweet syndrome [3]. CRMO is generally considered a benign disease; however, it can lead to persistent symptoms or sequela that can have an impact on quality of life [3].

For the most part, the pathophysiology of CRMO remains unclear, previously being compared to SAPHO syndrome (synovitis, acne, pustulosis, hyperostosis, and osteitis) or to an autoinflammatory disease. More recent genetic data suggest that CRMO may belong to a group of autoinflammatory diseases with osseous expression (Majeed syndrome, and PAPA syndrome (pyogenic sterile arthritis, pyoderma gangrenosum, and acne), and DIRA syndrome (deficiency of the IL-1R antagonist)) [3]. However, since anti-IL-1 therapy does not have the same level of improvements in CRMO as the aforementioned diseases, it points to that perhaps CRMO etiology is probably more multifactorial in nature [3]. Recent studies suggest a possible familial component to CRMO with the observation of the disease in siblings [5, 6]. Additionally, the Eurofever cohort found 14 of the 486 patients (2.8%) reported a CNO-affected relative [1]. As there have been reports of affected first-degree family members with other chronic inflammatory diseases, it posits the question of whether CNO may in fact have a genetic component. However, no definitive gene variants have been linked to CNO. A small German CRMO cohort study did identify a susceptibility locus at chromosome 18q21.3–22 that suggests a genetic component [7]. However, more recently a larger cohort study of 84 CRMO trios was not able to replicate the chromosome 18 CRMO susceptibility locus and therefore found no association between CRMO and inheritance of D18S60 [8].

Laboratory testing typically reveals an inflammatory syndrome, with high CRP and ESR, usually normal blood cell counts, and less likely positive HLA-B27 and ANA [3]. Imaging on average reveals three and a half structural lesions of the bone [3]. Bone biopsy histologic investigations range from inconclusive results or no significant change [1] to minimal fibrosis and inflammation, bone remodeling, and sclerosis [3]; lymphocytes are significantly the most frequently present inflammatory cells [1, 3].

There are three phenotypes of CRMO with distinct features and prognosis documented in the literature. The severe phenotype includes male patients with the multifocal form of CRMO, rare clavicle involvement, and exhibit evidence of inflammatory syndrome [3]. The mild phenotype is primarily females with a unifocal form of CRMO, commonly with clavicle involvement, sometimes with evidence of inflammatory syndrome, and rare extraosseous lesions [3]. The intermediate phenotype is also predominantly females with the multifocal form of CRMO, commonly exhibiting evidence of inflammatory syndrome and some with family history of associated disease and extraosseous lesions [3]. Severity of phenotype also correlates with prognosis. Prognostic factors of disease are associated with not achieving clinical remission at the patient's last medical visit, longer time from symptom onset to diagnosis, multifocal pattern before diagnosis, and male patients [3].

Medical treatment consists of NSAIDs as first-line therapy and then corticosteroids, methotrexate, sulfasalazine, bisphosphonates, anti-TNF*α* agents, and anti-IL-1R (anakinra), with efficacy rates reported in one case study to be 41% with sulfasalazine, 37.5% with methotrexate, 75% with bisphosphonates, and 89% with anti-TNF*α* agents [3]. DMARDs, particularly sulfasalazine, are considered in patients with frequent relapses or if NSAIDs need to be discontinued, and oral glucocorticoids are considered as a bridging agent for a limited period of time or in low-dose concomitant treatment [9]. Sequelae are possible even with treatment and can include localized bone deformation, especially of the clavicle, vertebral fractures, and growth retardation in children [3].

## 2. Case Presentation

**Figure 1:**
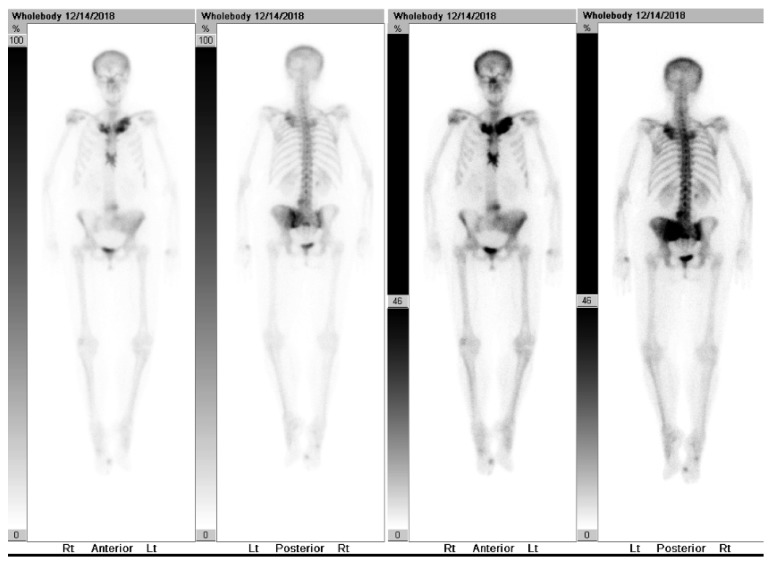
Nuclear bone scan performed on 12/14/2018 of CRMO. Imaging reveals increased uptake of the left side of pelvis and ileum best seen on posterior views, and the proximal sternum and bilateral medial clavicles best seen on anterior views.

**Figure 2:**
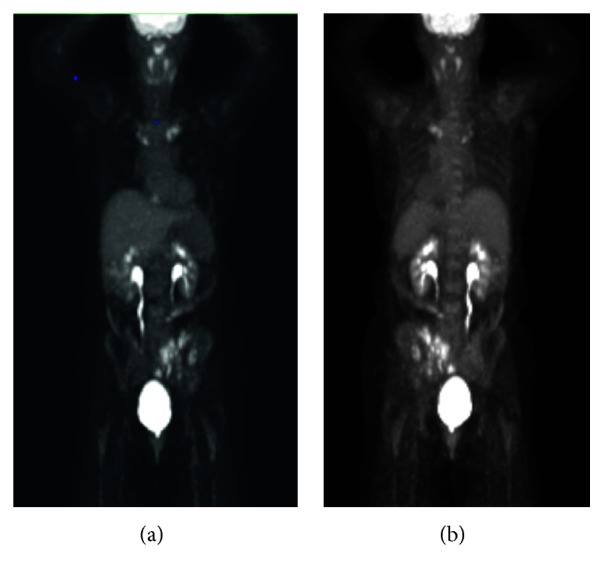
PET scan whole body performed on 1/9/2019. (a) Anterior view and (b) posterior view with hypermetabolism of the left side of pelvis and ileum and bilateral medial clavicles.

A 74-year-old female with a past medical history of a right radius fracture, right shoulder fracture, basal cell carcinoma of the right eye, chronic joint pain, and palmoplantar psoriasis originally presented to a local hospital's emergency department on 11/29/2018 after experiencing acute sharp pain in her left buttocks that radiated to her left foot after rising from a couch, with additional abdominal symptoms of bloating, belching, and nausea. CT abdomen and pelvis revealed an abnormal iliac bone and left sacrum with a combination of sclerotic and lytic areas that was initially suspected to be a malignant process, and she was referred to oncology at her local hospital. Additional workup included a mammogram on 12/27/2018 that revealed small asymmetry in the right breast and a bone scan on 12/14/2018 that revealed increased uptake and multifocal abnormal osseous activity in the left side of pelvis, ileum, proximal sternum, and bilateral medial clavicles (Figure 1). She underwent CT-guided bone biopsy at her local hospital on 12/28/2018 that revealed scant fragments of benign bone with no diagnostic abnormalities, but with concern that the biopsy was too superficial. A follow-up PET scan obtained on 1/9/2019 revealed hypermetabolism only in the bony sites previously identified by the bone scan without evidence of hypermetabolic visceral disease (Figure 2). For pain relief she was prescribed ibuprofen. After this additional workup, she was referred to our hospital's Orthopedic Surgery Department for further consultation and possible repeat biopsy.

**Figure 3:**
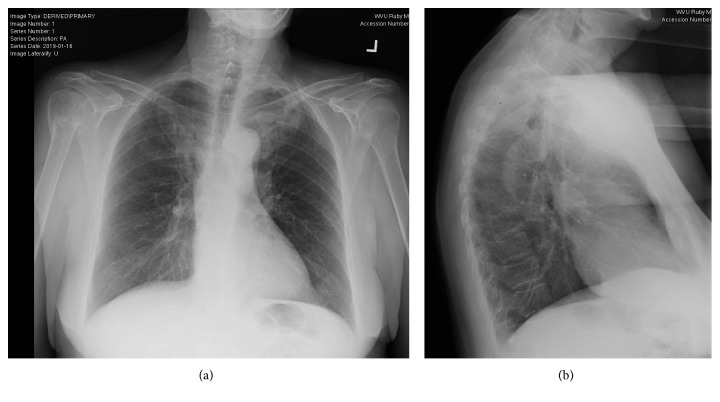
PA and lateral chest x-rays performed on 1/16/2019. (a) PA view with asymmetry at the left apex, which could relate to osseous structures, with questionable expansion of the proximal left first anterior rib, and (b) lateral view with questionable expansion of the inferior sternum.

**Figure 4:**
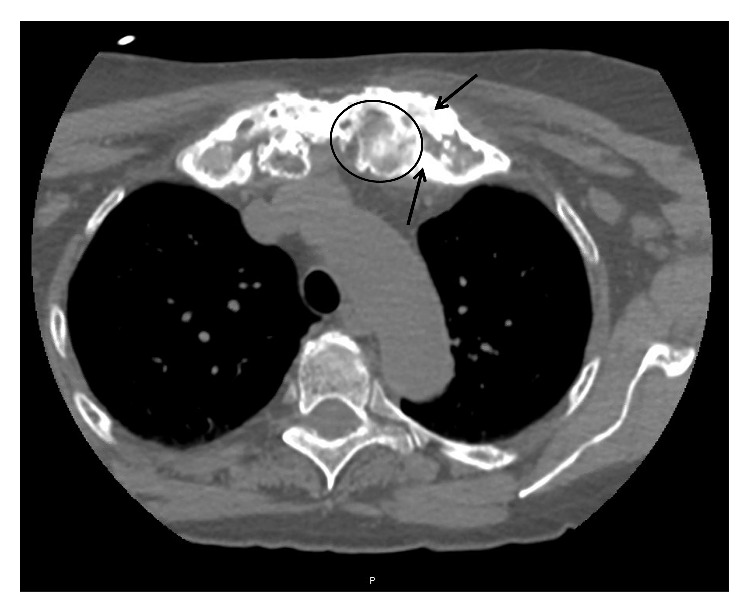
CT biopsy imaging axial body view with a slice thickness of 2.00 demonstrating lytic (example inside circle) and sclerotic lesions (examples of thickened bone pointed to with arrows) of the left medial clavicle and left first rib.

**Figure 5:**
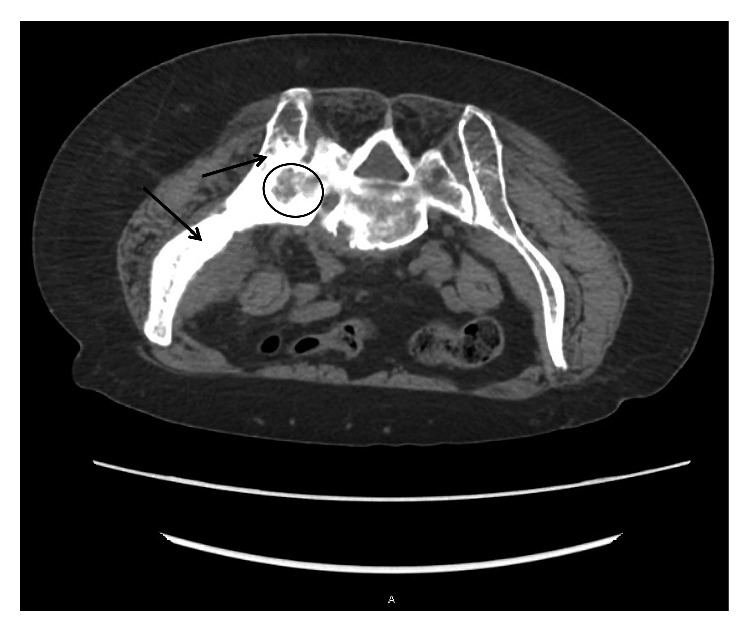
CT biopsy imaging axial body view with slice thickness 5.00 demonstrating lytic (example inside circle) and sclerotic lesions (examples of thickened bone pointed to with arrows) of the left sacrum and left ileum. Also, notice fusion of the left sacro-iliac joint. For reference, the patient is prone in this image as it was taken during the time of biopsy; therefore, the left ileum/sacrum is on the left of the image.

**Figure 6:**
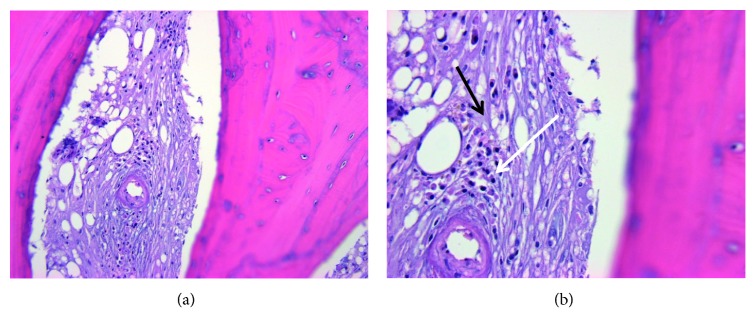
Bone biopsy of the left medial clavicle performed on 1/21/2019. (a) H&E 200x marrow fibrosis with plasma cell infiltrate. (b) H&E 400x marrow fibrosis (lighter purple color throughout indicated by black arrow) in place of a fatty marrow background with plasma cell infiltrate that contains eccentric nuclei (area indicated by white arrow) representing changes indicative of chronic inflammation.

**Figure 7:**
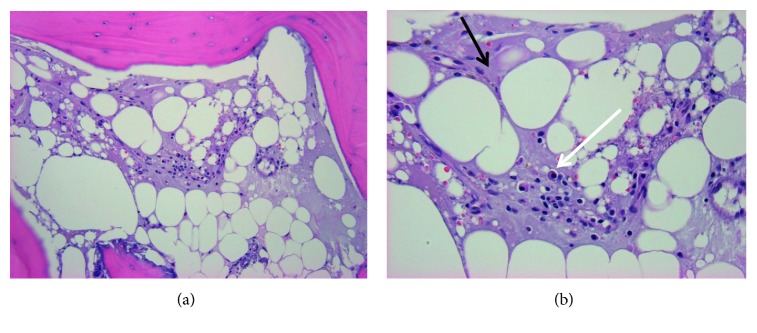
Bone biopsy of the left medial clavicle performed on 1/21/2019. (a) H&E 200x marrow fibrosis with plasma cell infiltrate. (b) H&E 400x marrow fibrosis (lighter purple color throughout indicated by black arrow) in place of a fatty marrow background with plasma cell infiltrate that contains eccentric nuclei (area indicated by white arrow) representing changes indicative of chronic inflammation.

Orthopedics at our institution initially evaluated the patient on 1/16/2019. She reported having left pelvic and hip pain with some recent improvement, 20 to 30 pound weight loss, and fatigue since the spring of 2018. Note that the patient was afebrile throughout this timeline. PA and lateral chest x-rays performed on 1/16/19 (Figure 3) demonstrated asymmetry at the apices with questionable osseous lesions, questionable expansion of the proximal left first anterior rib, and questionable expansion of the inferior sternum on the lateral view with resultant recommendation for chest CT for further characterization. Our institute's Interventional Radiology Department performed CT-guided biopsies on 1/21/19 of her left clavicle, first rib, sacrum, and ileum. CT imaging performed during the biopsies also commented on the lytic and sclerotic appearance of the lesions that were to be biopsied of the left medial clavicle and the left first rib (Figure 4), left sacrum, and left ileum (Figure 5). Pathology revealed dense bone with reactive changes and mild marrow fibrosis; changes noted to be consistent with a chronic inflammatory process such as chronic recurrent multifocal osteomyelitis (CRMO) (Figures 6 and 7); and no evidence of Paget's disease or neoplasm. Of note, the pathology was additionally sent to Johns Hopkins Reference Laboratories for review with resultant confirmation of CRMO. Bacterial, anaerobic, fungal, and AFB cultures failed to show any growth. Labs revealed elevated acute phase reactants—CRP (53.3 mg/L) and ESR (89 mm/hr), with thrombocytosis (platelets (452,000/*μ*L).

The patient was referred to our institute's Infectious Disease Department for evaluation given biopsy results revealing CRMO and was seen on 2/18/2019. The infectious disease specialist also confirmed the IR biopsy of the clavicle, first rib, sternum, and ileum which were consistent with chronic recurrent multifocal osteomyelitis. However, since CRMO occurs less commonly in adults, it was initially suspected the patient had SAPHO due to her remote history of palmoplantar psoriasis that was diagnosed by a dermatologist approximately 30 years ago and was treated for approximately ten years before resolving. She has not had recurrence in the last 20 years. Additionally, she has a history of multiple fractures after falls including her right wrist in August 2017 and right shoulder in January 2018. She also tested negative for syphilis and HIV.

Given the autoinflammatory nature of CRMO, the patient was referred to our institute's Rheumatology Department for further evaluation and management. CCP and RF were normal. CRP and ESR were still elevated; however, with improvement from prior values. The patient's symptoms are currently responding to the first-line therapy of NSAIDs. The patient is tolerating it well and currently prefers to continue this first-line treatment. Other possible future therapies like corticosteroids (as a bridge) and conventional DMARDs such as sulfasalazine can be considered if she fails NSAIDs or has intolerable side effects of NSAID treatment. Biologic DMARDs like TNFi (etanercept and Remicade) are also other possible options to consider. Bisphosphonates is another possible additive medication as a means of lowering pain and prevention of fractures in prophylactic dosing if the patient has no history of fractures and has a normal DEXA, or at a therapeutic dosing if the patient has osteoporosis on DEXA and/or a history of low impact fractures. Additionally, if the patient continues to experience left hip pain with impact on mobility then we will obtain MRI or PET-CT to ensure no active disease. MRI has proven sensitive in detecting bone edema during the early stages of inflammation and detecting more CNO lesions than x-rays or technetium bone scans [10].

## 3. Discussion

Nonbacterial osteitis (NBO) is a rare autoinflammatory bone disease with a presentation of acute or recurrent, unifocal or multifocal sterile bone lesions, and it is referred to as chronic recurrent multifocal osteomyelitis (CRMO) in cases where the bone lesions are recurrent and multifocal. CRMO is a diagnosis of exclusion (Algorithm 1), with differentials for our patient primarily being malignancy, chronic infectious osteomyelitis, and SAPHO. CRMO is a rare entity to be diagnosed in patients over 50 years of age and has no prior reported cases to our knowledge in elderly patients (age greater than 65 years) which makes this case the first of its kind.

Clinical features consistent with CRMO usually include a history of insidious onset, such as in our patient with years of bone pain of the affected sites, and are associated with swelling and tenderness over the affected bone sites. Clinical signs of bone inflammation include localized warmth, swelling, pain, and rarely erythema [11]. Pain typically occurs during the daytime but can also occur at night and can result in a limited range of motion of affected joints [4]. The severity and time course of the disease can vary with acute exacerbations and spontaneous remissions [4]. Our patient presented with a long history of pelvic and hip pain with a clear acute exacerbation at the beginning of this time course of events. Classically, the patient will present with a swollen clavicle [12]; can include extraosseous involvement of the skin in the form of palmoplantar pustulosis as our patient did approximately 30 years prior acneiform lesions or psoriasis; IBD particularly with Crohn's-like lesions has been reported in the literature in combination with CRMO and is suggested to represent “enteropathic CRMO” [12]. In adults, skin inflammation and mucocutaneous manifestations are more common than in children [1, 11] as was in our patient's case. The Eurofever cohort showed mucocutaneous manifestations of 41% in adults and 19% in children, primarily due to more cases of palmoplantar pustulosis. The metaphyses of the long bones, pelvis, and clavicles are the most commonly affected sites [3]. Our patient's area of involvement included the left pelvis, ileum, proximal sternum, and bilateral medial clavicles as noted on nuclear bone scan (Figure 1).

The diagnosis of CRMO is dependent on the exclusion of other diseases. Diagnosis is typically based on history, clinical exam, and radiological and histological findings. Imaging studies include plain radiography, computed tomography (CT), magnetic resonance imaging (MRI), PET scans, and nuclear bone scans. Plain x-rays can reveal periosteal reactions, soft tissue swelling, or lytic areas [12]. Radionucleotide bone scans can identify silent lesions in other bones (as foci of increased uptake) [12]. Whole body MRI using short tau inversion recovery (STIR) sequences can identify characteristic features of areas of bone marrow edema, is more sensitive than x-rays or bone scans to identify silent lesions without radiation, and can be used to monitor response to treatment [12]. MRI is generally the preferred imaging as it is more sensitive than radiographs and scintigraphy [4]. In a considerable number of patients, diagnostic imaging alone does not rule out malignancy; therefore, biopsy must be performed. Bone biopsies are typically sterile, with pathology demonstrating nonspecific inflammatory bone changes with bone remodeling and sclerosis [3].

Although bone biopsies remain necessary, some CRMO patients could avoid this invasive investigation according to the clinical score proposed by Jansson et al. to differentiate nonbacterial osteitis (NBO) from other bone lesions [13] in cases where the clinical history, examination, labs, and radiology are typical of the disease. Jansson et al. also proposed a set of diagnostic criteria in 2007, see Table 1 [14]. In using this NBO scoring system (Table 2) for our patient, she was positive for all the risk factors listed, with a total score of 63. This score indicates that based on her history, lab, and radiologic workup one could theoretically make the diagnosis of nonbacterial osteitis without having to perform an invasive biopsy. Previous cohort studies have also applied this score in efforts to demonstrate that the clinical score may be useful in routine practice for the management of CRMO in order to avoid unnecessary invasive investigations [3]. We propose that in a specific subgroup of patients who cannot undergo biopsy or who would be considered high risk to biopsy, a diagnosis of CRMO can be even considered in the elderly population based on the scoring system. Bone pain in patients can be a diagnostic challenge, and the differential diagnosis includes malignancy, benign bone tumors, bacterial osteomyelitis, and nonbacterial osteitis (see Algorithm 1, for a more inclusive list). The presence of nonbacterial bone lesions is typically a criterion for inflammatory disorders including SAPHO syndrome (synovitis, acne, pustulosis, hyperostosis, and osteitis), CRMO (chronic recurrent multifocal osteomyelitis), pustulotic arthro-osteitis, chronic sclerosing osteomyelitis, lymphoplasmacellular osteomyelitis, and other disorders [13].

There are no official treatment protocols based on randomized studies for CRMO. NSAIDs are the first-line treatment for CRMO and are highly effective as previously reported, improving pain in 40–80% of children who respond better to NSAIDs than adults [4, 9]. Our patient's pain was responsive to the first-line therapy of NSAIDs, specifically ibuprofen. Indomethacin and naproxen have been reported as effective in both primary lesions and for relapses as well [12]. For patients that do not respond to NSAIDs or for some that need to be discontinued on NSAIDs, steroids are typically considered next: methylprednisolone, oral prednisone, or hydrocortisone [12]. The use of sulfasalazine as DMARD is generally considered ineffective as single-agent therapy [12]. Bisphosphonates, specifically pamidronate, has been widely shown to reduce pain and improve function [12, 15]. Biological therapies have been increasing in use to treat CRMO, with literature documenting several case reports of success with infliximab, etanercept, and anakinra [12]. There seems to be a lack of controlled trials and studies to evaluate the second-line treatment options, especially based on phenotype, since choices of when to use each medication are often based on previous cases and studies in the literature.

## 4. Conclusions

What is important to note is that most of the literature on the subject of CRMO focuses on pediatric patients while there continues to be a limited number of adult patients and a paucity of elderly patients described in the literature with CRMO, including their course to diagnosis and treatment outcomes. While the disease is rare to be diagnosed in the adult and elderly, more studies are needed in order to determine the best method of diagnosis and treatment for the older patient. This is essential given the significant average delay in diagnosis of approximately one year [1]. While the diagnosis of CRMO is primarily based on exclusion of other differential diagnoses, history of recurrent bone pain, and the presence of sclerotic bone lesions, there do exist some diagnostic tools such as the diagnostic criteria proposed by Jansson et al. [14] (Table 1) and score coefficient calculation by Jansson et al. [13] (Table 2). However, based on this necessity of exclusion of other differential diagnosis, bone biopsy remains mandatory in particular in adult patients. It is important to improve the clinical understanding of CRMO by the documentation of more cases, route of diagnosis, and treatment choices made in order to help develop clearer diagnostic and treatment guidelines.

## Figures and Tables

**Algorithm 1:**
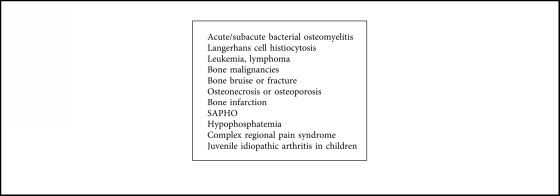
Differential diagnosis of chronic recurrent multifocal osteomyelitis (CRMO).

**Table 1:** Diagnostic criteria of NBO/CRMO.

Major diagnostic criteria	Minor diagnostic criteria
1. Radiologically proven osteolytic/sclerotic bone lesion	A. Normal blood count and good general state of health
2. Multifocal bone lesions	B. CRP and ESR mildly to moderately elevated
3. Palmoplantar pustulosis (PPP) or psoriasis	C. Observation time longer than 6 months
4. Sterile bone biopsy with signs of inflammation and/or fibrosis, sclerosis	D. Hyperostosis
	E. Associated with other autoimmune diseases apart from PPP or psoriasis
	F. Grade I or II relatives with autoimmune or autoinflammatory disease or with NBO

Diagnostic criteria from Jansson et al. Diagnosis confirmed with 2 major criteria or 1 major and 3 minor criteria [14].

**Table 2:** Score coefficient calculation for NBO diagnosis.

Risk factor	Score coefficient
Normal blood cell count	13
Symmetric lesions	10
Lesions with marginal sclerosis	10
Normal body temperature	9
Vertebral, clavicular, or sternal lesions	8
Radiologically proven lesions ≥2	7
CRP ≥1 mg/dl	6
Total clinical score	63

Optimal multivariable logistic regression model for calculating the clinical score for a diagnosis of nonbacterial osteitis from Jansson et al. [14]. Risk factors were included if the regression coefficient was statistically significant (*P* < 0.05). The total clinical score is the sum of score coefficients not equal to zero, with a range of 0–63.
